# Prospective Associations Between Parenting Styles and Adolescents’ Social–Emotional Competence: A Cross-Lagged Network Analysis

**DOI:** 10.3390/bs16101869

**Published:** 2026-10-10

**Authors:** Xiaomin Yuan, Xingyi Wang, Xinpeng Li, Shuguang Zhang

**Affiliations:** 1 School of Education Science, Shanxi University, Taiyuan 030006, China; yuanxiaomin@sxu.edu.cn (X.Y.); wangxingyi@sxu.edu.cn (X.W.); 2 Yuci Affiliated School of Shanxi Normal University, Jinzhong 030600, China; 18235133136@163.com; 3 Department of Psychology, Shanxi University, No. 92, Wucheng Road, Xiaodian District, Taiyuan 030006, China

**Keywords:** social–emotional competence, parenting styles, parental autonomy support, parental psychological control, cross-lagged panel network

## Abstract

Adolescence involves substantial developmental changes, with social–emotional competence (SEC) serving as an important protective factor. However, how parenting styles are prospectively associated with adolescents’ SEC in the Chinese cultural context remains unclear. A two-wave longitudinal study was conducted among 1167 middle school students in China (52.4% male; baseline M_age_ = 13.16 years, SD = 0.93). Using longitudinal data and a cross-lagged panel network analysis (CLPN), we examined the temporal relationships among parental autonomy support, psychological control, and SEC. Opinion exchange showed a positive prospective association with later self-management. Adolescent self-management showed a limited adolescent-to-parent prospective association, whereas responsible decision-making showed relatively prominent incoming and outgoing prospective connectivity and may warrant further investigation in confirmatory and intervention research. These findings highlight the potential relevance of parent–adolescent communication and emotional support for adolescents’ social–emotional functioning.

## 1. Introduction

Adolescence is a critical developmental stage during which teenagers strive for greater independence while simultaneously navigating increasingly complex emotional, interpersonal, and decision-making challenges (P. J. C. Costa et al., 2025). Social–emotional competence (SEC) serves as a vital developmental resource for coping with identity exploration, emotional fluctuations, interpersonal restructuring, and growing autonomy, laying a solid foundation for adolescents to meet these needs (Collie & Ryan, 2025; P. J. C. Costa et al., 2025; Mahoney et al., 2021). Consequently, SEC acts as a crucial protective buffer that enables students not only to navigate these transitional periods successfully but also to thrive holistically.

The family constitutes the core microsystem of adolescent development, with recurrent proximal interactions between parents and children serving as a key precursor to capacity formation (Bronfenbrenner, 1979; Baptist & Hamon, 2022). Current research predominantly focuses on the relationship between parenting styles and adolescents’ externalizing or internalizing behaviors (e.g., Brenning et al., 2019), while overlooking the connection between parenting approaches and positive psychological outcomes. As components of parenting styles, autonomy support and psychological control not only influence adolescent psychological outcomes through positive or negative effects, but also manifest differently in psychological outcomes (S. Costa et al., 2016). According to the Self-Determination Theory (SDT; Ryan & Deci, 2017), autonomy support fosters intrinsic motivation, self-regulation, and positive emotional development. Conversely, psychological control (Barber, 1996) undermines psychological autonomy and is associated with emotional dysregulation and poorer social functioning. Although parental autonomy support and psychological control are negatively correlated, these two behavioral approaches can coexist within the same family context and should not be simplistically viewed as opposing extremes on a continuum (Hauser Kunz & Grych, 2013; Soenens & Vansteenkiste, 2010). Therefore, it is essential to integrate them into a unified model to investigate their specific impacts on adolescents’ SEC.

However, few longitudinal studies have simultaneously modeled autonomy support and psychological control as independent constructs within the same framework, leaving their relative contributions to adolescent SEC and their dynamic interactions unclear. Moreover, traditional research typically focuses on linear relationships between variables, potentially oversimplifying the complex interconnections between these concepts and limiting our understanding of SEC’s multidimensional nature as well as the intricate, mutually influential relationship between autonomy support and psychological control.

The network perspective models the measured facets as an interconnected system. A cross-lagged panel network (CLPN) estimates conditional prospective associations among nodes while accounting for the other included nodes and their prior levels (Borsboom & Cramer, 2013; Borsboom et al., 2021/2022). These directed temporal associations can help identify candidate pathways for further study; we therefore used longitudinal surveys and a network analysis of Chinese adolescents to examine dimension-specific prospective associations among parental autonomy support, parental psychological control, and SEC, with the goal of identifying the family interaction factors associated with a specific SEC.

### 1.1. Social–Emotional Competence as a Differentiated System

SEC refers to a set of interconnected abilities that enable individuals to recognize and understand their own and others’ emotions, regulate emotions and behaviors, build and maintain relationships, and make responsible decisions (Mantz et al., 2018). Treating the SEC as a single overall score can easily obscure the distinct underlying causes of varying abilities and their interactions. By modeling skills at the facet level, Guo et al. (2023) identified the key skills for different outcomes and found significant variation in facets’ predictive utility even within the same domain. A network analysis demonstrated that social–emotional skills do not exhibit a homogeneous structure; their centrality and connectivity patterns vary across age groups and cultural contexts (Huo & Ning, 2025). In the Delaware framework (CASEL, 2020; Mantz et al., 2018), responsible decision-making involves evaluating consequences and selecting constructive actions; relationship skills support communication, cooperation, and relationship maintenance; self-management concerns the regulation of emotion and behavior in pursuit of goals; and social awareness involves recognizing others’ perspectives and social cues. Self-management enables adolescents to inhibit impulses and make decisions; social awareness provides information for understanding others and contexts; relationship skills translate comprehension and regulation into effective interactions; while responsible decision-making integrates consequence assessment, self-control, and social norms. These skills are distinct yet interconnected. For example, responsible decision-making requires self-regulation and sensitivity to others’ circumstances, while healthy relationships depend on the synergy between social awareness and behavioral control. Treating SEC as a differentiated system has two advantages. Conceptually, it avoids assuming that a family process must affect every social–emotional capacity equally. Empirically, it permits the identification of capacities that are more central or more responsive within a larger developmental network.

### 1.2. Parenting Styles and Adolescents’ Social–Emotional Competence

Parental autonomy support refers to parenting support provided by parents through encouraging independence, offering emotional support. Parental psychological control refers to a series of intrusive parenting strategies designed to compel children to think, behave, or feel in ways that parents approve of (Q. Wang et al., 2007). Numerous cross-sectional studies have shown that autonomy support is positively correlated with adolescents’ self-awareness, social skills, and emotional regulation, whereas psychological control exhibits the opposite pattern (e.g., Cui et al., 2014).

SDT (Ryan & Deci, 2017) posits that autonomy, competence, and belonging are fundamental psychological needs across cultures, and their fulfillment serves as the intrinsic motivation for developing SEC. When the environment supports these needs (autonomy support style), individuals are more likely to cultivate autonomous motivation, internalize norms, and develop effective self-regulation; conversely, when the environment impedes them (psychological control style), controlled motivation, psychological distress, and adaptation difficulties are more likely to emerge (Goagoses et al., 2023; Y. Wang & Guo, 2024; Bradshaw et al., 2025).

Furthermore, according to social cognitive theory (Bandura, 1986), adolescents learn emotional regulation by observing how their parents express and manage emotions, responding to specific parental reactions to their emotions, and experiencing the overall emotional atmosphere within the family (Morris et al., 2007; Bandura, 1986). Autonomy support—characterized by listening, explanation, and negotiation—embodies respect, empathy, and constructive conflict resolution; adolescents not only receive emotional validation but also have opportunities to practice expression, regulation, and decision-making. Conversely, guilt-inducing behaviors, the withdrawal of affection, and the suppression of dissent create an unpredictable or conditionally accepting emotional environment that predisposes adolescents to depressive, rumination-driven, avoidance-oriented, or impulsive responses.

### 1.3. Potential Bidirectional Dynamic Interaction

Adolescents are also key agents influencing parent–child relationships, whose abilities and behaviors may affect parents’ continuous adjustments to their choice frameworks, communication styles, and levels of control. The existing bidirectional evidence identifies at least three types of child effects. First, adaptation difficulties may be associated with increased control. Deviant peer interactions among Chinese adolescents predict heightened parental psychological control (Yu et al., 2021), while low subjective well-being predicts subsequent increases in psychological control (Deng et al., 2024). Second, positive capabilities may foster autonomy. Prosocial behaviors in adolescents predict subsequent autonomy support from mothers (van Meegen et al., 2024), whereas strong autonomy predicts reduced parental psychological control (J. Wang et al., 2023). Third, emotional and capability states alter interactions over shorter timeframes. Daily positive emotions and autonomy supportive interactions reinforce each other, whereas negative emotions often precede controlling interactions (van der Kaap-Deeder et al., 2023). When adolescents demonstrate strong self-management skills, reliable decision-making abilities, and effective interpersonal skills, parents are more likely to trust their capabilities and grant greater autonomy. When adolescents exhibit maladaptive or risky behaviors, parents may adopt protective interventions; however, intrusive control can frustrate needs, impair adjustment mechanisms, and potentially lead to social withdrawal. Gao et al. (2021) separately examined love withdrawal, guilt induction, and shame control, and the results demonstrated that better emotional regulation in adolescents predicted a decline in all three types of maternal psychological control measures one year later. Notably, the relative strength of the child effect versus the parent effect varies over time. Specifically, immediate interactions, changes over months, and developments over years reflect distinct developmental processes.

### 1.4. Facet-Specific Associations Between Parenting and Social–Emotional Competence

Autonomy support is represented by choice making and opinion exchange. Choice making gives adolescents structured opportunities to exercise agency and assume ownership of behavior. Opinion exchange requires adolescents to articulate a position, listen to alternatives, negotiate disagreement, and revise a course of action (Q. Wang et al., 2007). The two dimensions of autonomy support provide distinct learning opportunities. The choice dimension confers agency for direct support, responsibility assumption, and standard internalization, which theoretically correlates more closely with responsible decision-making and self-management, whereas opinion exchange directly involves communication skills, perspective coordination, and behavioral reflection, thereby potentially influencing SEC across multiple interconnected dimensions. Through equitable communication, parents provide adolescents with forward-looking guidance in social contexts, enabling them to develop social skills and self-management abilities during parent–child interactions. Under the framework of Social Cognitive Theory (Bandura, 1986), this communication approach also facilitates cognitive restructuring, helping adolescents internalize family-derived social experiences and guidance as intrinsic value principles that benefit their future social engagement and adaptation.

With the aim of purposefully altering adolescents’ internal cognition, psychological control operates through various mechanisms, including guilt induction, love withdrawal, and authority assertion (Barber, 1996; Q. Wang et al., 2007).

Guilt induction pressures compliance by making the adolescent feel responsible for parental disappointment; love withdrawal makes affection or relational security appear conditional on obedience; and authority assertion suppresses disagreement through parental power. All three may frustrate autonomy, but they need not have identical consequences. Guilt induction may foster self-doubt and externally regulated behavior. Authority assertion may limit opportunities to express, defend, and reconsider one’s views.

Love withdrawal may be especially relevant to relational security and decisions made under interpersonal pressure. According to the Self-Determination Theory (SDT), this strategy links familial security with obedience, directly undermining adolescents’ fundamental needs—not only hindering their autonomy but also eroding their sense of belonging. The attachment perspective (Bowlby, 1969; Freeman & Brown, 2001) further explains why the withdrawal of love can particularly impair relational competence and responsible decision-making. When relational security depends on obedience, adolescents may prioritize avoiding the loss of affection over making decisions grounded in internalized values.

### 1.5. Parenting and Social–Emotional Competence in the Chinese Cultural Context

The meanings and functions of parenting practices should also be considered within their cultural context. Chinese family socialization has traditionally emphasized respect for parental authority. These styles have contributed to debate regarding whether some forms of parental control may be interpreted differently in Chinese families than in more individualistic cultural contexts (Q. Wang et al., 2007). At the same time, psychological control should not be assumed to have uniform meanings or consequences simply because it occurs within a particular cultural context. Contemporary Chinese parenting may incorporate both autonomy-supportive and controlling practices. For example, Xu et al. (2024) reported changes in both autonomy support and psychological control among urban Chinese parents between 2013 and 2021, suggesting that these practices may coexist as parenting styles change. Moreover, cultural context may also be relevant to the organization of SEC themselves, as their centrality and connectivity have been found to vary across cultural settings (Huo & Ning, 2025). Accordingly, the Chinese context provides an important setting in which to examine whether autonomy support and psychological control show differentiated associations with adolescents’ SEC. The present study will examine facet-specific associations within a contemporary Chinese adolescent sample.

### 1.6. Network Analysis

To capture the complex, multi-dimensional system encompassing autonomy support, psychological control, and SEC, a network analysis provides a suitable tool for examining this complexity (Borsboom & Cramer, 2013; Borsboom et al., 2021/2022). From a network analysis perspective, cognition, emotion, and behavior do not exist in isolation, but rather shape the characteristics of psychological phenomena through interrelated processes within a holistic system. Specifically, these observed variables form a dynamic relational structure through their interactions with one another. Within this network, an individual’s specific psychological state is jointly determined by the direct connections between these variables.

Centrality in contemporaneous networks describes how strongly a node is connected to other nodes within the same measurement occasion, but it does not establish temporal direction or causal leverage (Borsboom & Cramer, 2013). CLPN enables researchers to estimate conditional prospective associations while distinguishing between central nodes with a high output (influencing other nodes) and those with a high input (being influenced by other nodes). Nodes exhibiting high-output centrality drive the progression of psychological outcomes or diseases, making them potential targets for clinical intervention (Epskamp et al., 2018).

Traditional longitudinal models and network approaches address related but distinct research questions. Standard cross-lagged panel models are designed to estimate autoregressive and cross-lagged associations among repeatedly measured constructs (Hamaker et al., 2015), whereas latent growth models primarily characterize the within-person trajectories of change and between-person differences in those trajectories (Grimm & Ram, 2018). Although these approaches are valuable for examining relations or developmental trajectories at the construct level, aggregating multidimensional constructs may obscure heterogeneous associations among their specific facets. In contrast, a network analysis represents multiple measured facets as an interconnected system and allows their unique associations to be examined simultaneously (Borsboom & Cramer, 2013; Borsboom et al., 2021/2022).

### 1.7. The Present Study

Based on the theoretical and empirical evidence reviewed above, the present study integrates parental autonomy support, psychological control, and SEC facets within a two-wave CLPN to examine short-term facet-specific prospective associations among Chinese adolescents. The present study aimed to (1) characterize the conditional associations among parenting and SEC facets within the contemporaneous networks; (2) examine facet-specific prospective associations from parenting dimensions to subsequent SEC; (3) examine whether SEC facets also showed prospective associations with subsequent parenting dimensions; and (4) identify components characterized by relatively prominent cross-domain connectivity and incoming or outgoing prospective associations. Accordingly, we propose the following hypotheses:

**H1a.**  *In the contemporaneous networks, parental autonomy-supportive practices (choice making and opinion exchange) will show positive cross-domain associations with adolescents’ SEC facets.*

**H1b.**  *In the contemporaneous networks, parental psychological control (guilt induction, love withdrawal, and authority assertion) will show negative cross-domain associations with adolescents’ SEC facets.*

**H2a.**  *Higher parental choice making at W1 will be positively associated with all four SEC facets at W2.*

**H2b.**  *Higher parental opinion exchange at W1 will be positively associated with all four SEC facets at W2.*

**H3a.**  *Higher guilt induction at W1 will be negatively associated with subsequent self-management and responsible decision-making at W2.*

**H3b.**  *Higher love withdrawal at W1 will be negatively associated with all four SEC facets at W2.*

**H3c.**  *Higher authority assertion at W1 will be negatively associated with subsequent responsible decision-making at W2.*

**H4.**  *Higher self-management and responsible decision-making at W1 will be prospectively associated with greater subsequent autonomy-supportive parenting and lower subsequent psychological control at W2.*

## 2. Methods

### 2.1. Procedures and Participants

The study comprised two waves of longitudinal surveys of adolescents three months apart, recruiting a sample of middle school students in two cities in Shanxi Province, China. Data were collected at two time points: near the end of the first semester and at the beginning of the second semester of the 2025–2026 academic year, the interval intentionally spanned a period in which family routines and parent–adolescent contact may change. Participants were included in the longitudinal analysis if they provided valid questionnaire data and could be successfully matched across both waves. Questionnaires were excluded when responses failed the two attention-check items, contained substantial blank responses, or was shown to be clearly patterned. Participants with valid W1 data who were not successfully followed at W2 were treated as attrition cases and were not included in the final two-wave analytic sample. At Wave 1 (W1), 1321 adolescents participated. Of these participants, 1167 were successfully followed and matched at W2, constituting the final two-wave analytic sample (retention rate = 88.3%). Among the 1167 retained participants, the mean age at W1 was 13.16 years (SD = 0.93), and 612 were boys (52.4%). In the final two-wave analytic sample, paternal educational attainment at primary school or below was 8.14%, junior high school 52.53%, high school 27.34%, and college or above 12.00%; the corresponding maternal percentages were 7.54%, 46.62%, 30.93%, and 14.91%.

This research was approved by the Ethics Review Board of Shanxi University (Approval Number: SXULL2025128).

Attrition analyses were conducted to compare participants who were successfully followed across both waves (n = 1167) with those who participated at W1 but were not retained at W2 (n = 154). Benjamini–Hochberg false-discovery-rate correction was applied across the baseline comparisons. Compared with retained participants, participants lost to follow-up were slightly older at W1 (M = 13.42, SD = 1.05 vs. M = 13.16, SD = 0.93), Welch’s t(179.53) = −2.82, *p* = 0.019, Hedges’ g = −0.27, and reported higher levels of love withdrawal (M = 2.27, SD = 1.08 vs. M = 1.98, SD = 1.00), Welch’s t(190.24) = −3.25, *p* = 0.015, Hedges’ g = −0.30. Gender distribution also differed between the two groups, χ^2^(1) = 9.01, *p* = 0.015, Cramér’s V = 0.083. No significant differences were observed for the other eight baseline study variables (all adjusted *p*s ≥ 0.152). Overall, although some evidence of selective attrition was observed, the corresponding effect sizes were small.

### 2.2. Measures

#### 2.2.1. SEC

The Delaware Social-Emotional Competency Scale (Mantz et al., 2018) was used to assess responsible decision-making, relationship skills, self-management, and social awareness on a 4-point scale. Reliability was good across waves (Cronbach’s *α* for total SEC = 0.735–0.761; responsible decision-making: *α* = 0.761–0.764; relationship skills: *α* = 0.701–0.726; self-management: *α* = 0.745–0.787; social awareness: *α* = 0.701–0.743). The test–retest reliability was acceptable (Intraclass Correlation Coefficient, ICC = 0.721–0.793).

#### 2.2.2. Parenting Styles

The Psychological Control Scale and Psychological Autonomy Support Scale developed by Q. Wang et al. (2007) were used, which include the dimensions of choice making, opinion exchange, guilt induction, love withdrawal, and authority assertion, and are scored on a 5-point scale. Reliability was good at all waves (Cronbach’s *α* for Parental Psychological Autonomy Support: 0.868–0.877; Choice Making: *α* = 0.731–0.826; Opinion Exchange: *α* = 0.711–0.856; Parental Psychological Control: *α* = 0.929–0.940; Guilt Induction: *α* = 0.876–0.890; love withdrawal: *α* = 0.858–0.897; authority assertion: *α* = 0.787–0.819). The test–retest reliability was acceptable (ICC = 0.714–0.748).

### 2.3. Longitudinal Measurement Invariance

Longitudinal measurement invariance was examined separately for SEC, parental autonomy support, and psychological control using robust maximum likelihood estimation. Residuals of corresponding items were allowed to correlate across waves. Configural, metric, scalar, and strict invariance models were tested sequentially. Invariance was considered supported when ΔCFI was no lower than −.010 and ΔRMSEA was no greater than 0.015; ΔSRMR thresholds were 0.030 for metric invariance and 0.010 for scalar and strict invariance. The configural models showed acceptable fit for SEC (CFI = 0.935, TLI = 0.915, RMSEA = 0.040, SRMR = 0.046), parental autonomy support (CFI = 0.963, TLI = 0.956, RMSEA = 0.044, SRMR = 0.029), and parental psychological control (CFI = 0.906, TLI = 0.895, RMSEA = 0.065, SRMR = 0.045). Changes in model fit remained within the recommended criteria after imposing equality constraints on factor loadings, item intercepts, and residual variances (|ΔCFI| ≤ 0.0041, ΔRMSEA ≤ 0.0012, and ΔSRMR ≤ 0.0030; see Supplementary Table S2 for full results).

### 2.4. Statistical Analysis

SPSS Version 26 was used for descriptive statistical analyses. Participants who were lost to follow-up at Wave 2 were treated as attrition cases and were not imputed using the Expectation-Maximization (EM) algorithm. The longitudinal analytic dataset was first constructed by matching participants who provided data at both W1 and W2. Within this matched sample, item-level missing responses were imputed using the EM algorithm.

Dimension scores were calculated before network estimation. The four SEC dimension scores were formed by summing the three items assigned to each dimension. The two parental autonomy support dimension scores and the three psychological control dimension scores were calculated by averaging their corresponding items. All nine dimension scores were subsequently standardized as z scores.

The contemporaneous networks were estimated separately at each wave from the nine standardized dimension scores using the extended Bayesian information criterion graphical least absolute shrinkage and selection operator (EBIC graphical LASSO). For the temporal CLPN, each Wave 2 node was regressed on all nine Wave 1 nodes, with age, gender, paternal educational attainment, and maternal educational attainment included as covariates. Covariate effects were accounted for when estimating the temporal coefficients but were excluded from the network visualization and centrality calculations.

Network analyses were conducted in R version 4.5.0 using the bootnet, glmnet, networktools, and qgraph packages (Epskamp et al., 2018; Jones, 2020). Graphical Gaussian models were estimated using graphical LASSO regularization, and the optimal network was selected based on the EBIC (*γ* = 0.5; Zainal & Newman, 2023/2024).

First, contemporaneous networks were estimated separately for W1 and W2. Within the network, nodes that are closer to each other and connected by thicker lines indicate a stronger association. We calculated centrality measures for both networks to determine the importance of each node (i.e., its degree of association with all nodes in the other cluster or construct). These included edge strength and bridge Expected Influence (bridge EI; the sum of edge weights from a single node to all nodes in other variable component clusters, including bridge EI1 and bridge EI2 for W1 and W2). A higher bridge EI indicates that a node has a stronger influence on other variable component clusters.

Second, a CLPN from W1 to W2 was estimated. Directed edges represent conditional prospective regression associations after accounting for the other included W1 nodes and modeled covariates. The out-expected influence (outEI) and in-expected influence (inEI) measures were calculated as centrality indicators, representing the total influence exerted by a node on other nodes and the total influence received by that node from other nodes, respectively.

Network stability was evaluated using case-dropping bootstrap with 1000 bootstrap resamples to obtain 95% confidence intervals (CIs). Correlation stability (CS) coefficients were calculated for edge weights, bridge expected influence, outEI, and inEI (Epskamp et al., 2018). A CS coefficient >0.25 was considered acceptable, whereas a value >0.50 indicated good stability.

Power and sensitivity analyses were conducted to evaluate the adequacy of the analytic sample for the cross-lagged network analysis. With an analytic sample of *N* = 1167, the analysis had 99.8% power to detect a small incremental effect (*f*^2^ = 0.020, α = 0.05). A sensitivity analysis further indicated that the minimum effect detectable with 80% power was *f*^2^ = 0.00675, corresponding to a partial *r* = 0.082. After accounting for predictor correlations and outcome-specific residual variances, the median minimum detectable standardized path coefficient was |β| = 0.099 (range = 0.068–0.145).

To compare global network characteristics across waves, we calculated network density, global strength (the sum of absolute edge weights), the Pearson correlation of corresponding edge weights, and the Jaccard overlap of nonzero edges. We then conducted a paired Network Comparison Test with 1000 permutations to examine invariance in global strength and network structure. Edge-specific tests were adjusted using the Holm procedure. To quantify the predictive performance of the temporal model, we used 20 repetitions of 10-fold cross-validation and calculated out-of-sample R^2^ for each Wave 2 node.

## 3. Results

### 3.1. Descriptive Statistics

Descriptive statistics for the study variables are presented in Table 1, whereas correlations among demographic characteristics and study variables across both waves are provided in Supplementary Table S1.

### 3.2. Contemporaneous Networks

**Figure 1:**
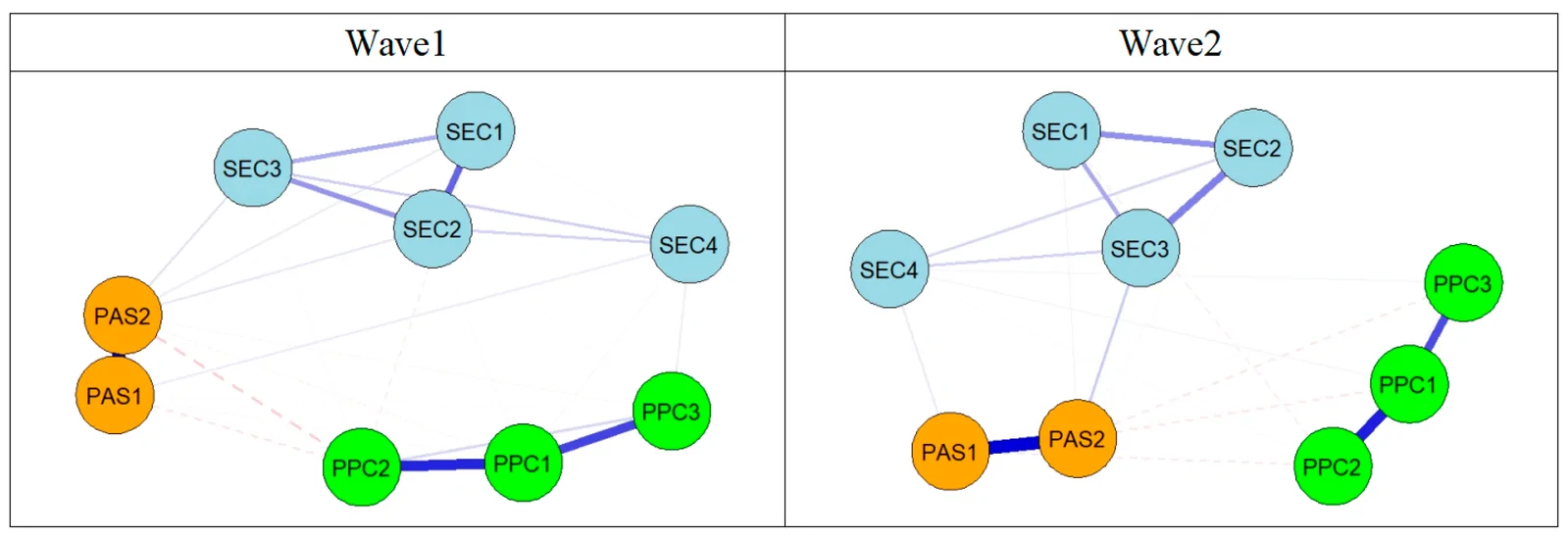
The contemporaneous network of W1 and W2. SEC1: responsible decision-making; SEC2: relationship skills; SEC3: self-management; SEC4: social awareness; PAS1: choice making; PAS2: opinion exchange; PPC1: guilt induction; PPC2: love withdrawal; PPC3: authority assertion. Blue nodes indicate SEC, orange nodes reflect parent autonomy support, and green nodes denote parent psychological control. Blue lines indicate positive relations, whereas red dotted lines signal negative relations, and line thickness and boldness reflect strength of associations.

Figure 1 shows the W1 and W2 contemporaneous networks, and Table 2 reports the non-zero regularized edge weights. Within the network cluster, the strong non-zero estimated edge is the negatively signed responsible decision-making-love withdrawal (rSEC1-PPC2 = −0.050). Positively correlated edges include self-management-opinion exchange (rSEC3-PAS2 = 0.121), social awareness -choice making (rSEC4-PAS1 = 0.058), relationship skills—opinion exchange (rSEC2-PAS2 = 0.050). Among the SEC, parental autonomy support, and parental psychological control components, those with the highest expected bridging effects were social awareness (SEC4; bridge EI1 = 0.144, bridge EI2 = 0.149), opinion exchange (PAS2; bridge EI1 = 0.027, bridge EI2 = 0.076), and love withdrawal (PPC2; bridge EI1 = −0.266, bridge EI2 = −0.118). The negative bridge expected influence of love withdrawal indicates that its connections with nodes in other communities were negative on balance. With respect to the proposed hypotheses, H1a was partially supported; H1b was partially supported.

Case-dropping bootstrap analyses indicated a good stability of the contemporaneous networks, with CS coefficients of 0.75 for both the edge strength and bridge expected influence at W1 and W2. The 95% confidence intervals (CIs) for the edge and bridging EI were relatively narrow, suggesting that the edges and the bridge EIs were estimated with good precision (see Supplementary Figure S1).

### 3.3. Temporal Networks

**Figure 2:**
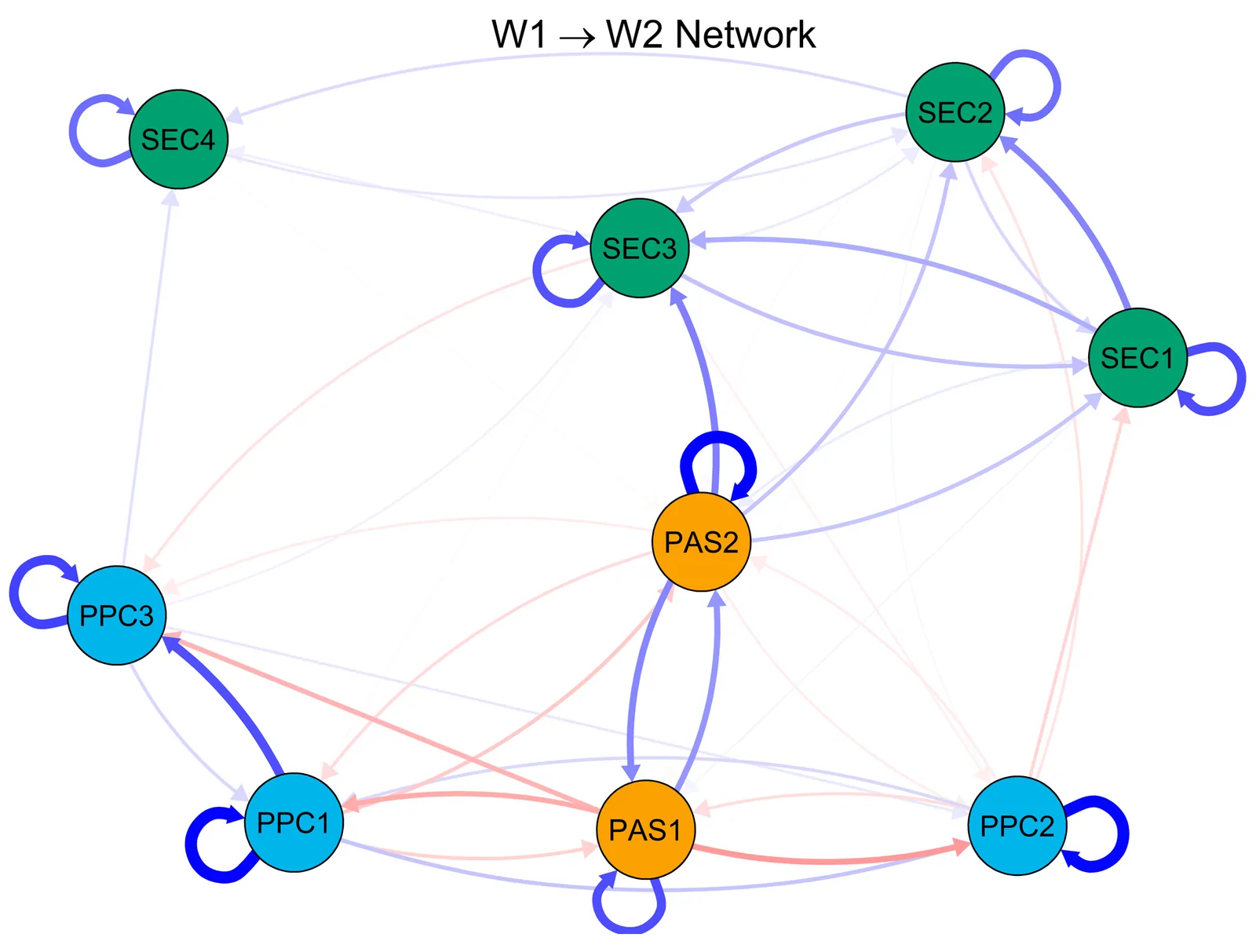
Wave 1 to Wave 2 temporal network of parenting styles and SEC components. SEC1: responsible decision-making; SEC2: relationship skills; SEC3: self-management; SEC4: social awareness; PAS1: choice making; PAS2: opinion exchange; PPC1: guilt induction; PPC2: love withdrawal; PPC3: authority assertion. Green nodes indicate SEC, dark yellow nodes reflect parent autonomy support, and blue nodes denote parent psychological control. Blue lines indicate positive relations, whereas red dotted lines signal negative relations, and line thickness and boldness reflect strength of associations.

**Figure 3:**
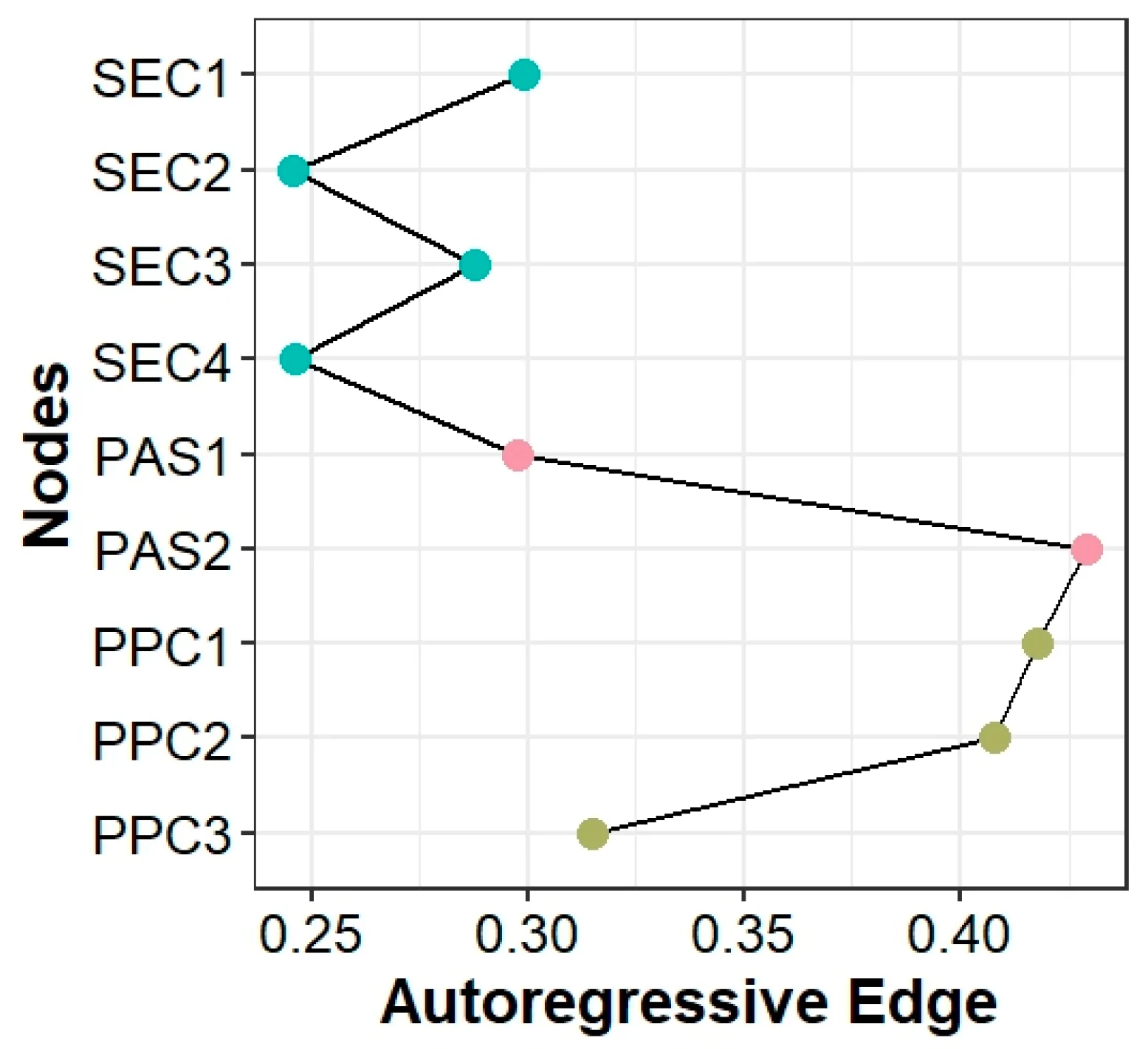
Autoregressive edge weights for the nine nodes from Wave 1 to Wave 2. SEC1: responsible decision-making; SEC2: relationship skills; SEC3: self-management; SEC4: social awareness; PAS1: choice making; PAS2: opinion exchange; PPC1: guilt induction; PPC2: love withdrawal; PPC3: authority assertion. Blue dots indicate SEC, pink dots represent parental autonomy support, and green dots denote parental psychological control.

Figure 2 shows the CLPN, with arrows relaying the temporal associations of the edges within and across constructs. The largest autoregressive coefficients were observed for opinion exchange (PAS2; β = 0.429), guilt induction (PPC1; β = 0.418), and love withdrawal (PPC2; β = 0.408; Figure 3).

Table 3 shows the non-zero directed edges within and across constructs or clusters. Across domains, a higher W1 opinion exchange (PAS2) showed a positive prospective association with higher W2 self-management (SEC3; β = 0.218, 95% CI [0.104, 0.376]). In the adolescent-to-parent direction, higher W1 self-management (SEC3) showed a small negative prospective association with W2 authority assertion (PPC3; β = −0.050, 95% CI [−0.099, −0.003]). Positive point estimates were also observed from PAS2 to W2 relationship skills (SEC2; β = 0.101, 95% CI [0.000, 0.270]) and responsible decision-making (SEC1; β = 0.098, 95% CI [0.000, 0.254]); however, because their bootstrap CIs included zero, these paths were not considered clearly distinguishable from zero. Similarly, the negative point estimates from W1 guilt induction (PPC1) to W2 opinion exchange (PAS2; β = −0.091, 95% CI [−0.167, 0.000]) and from W1 love withdrawal (PPC2) to W2 responsible decision-making (SEC1; β = −0.071, 95% CI [−0.206, 0.000]) were interpreted cautiously because their CIs included zero. Overall, the cross-domain prospective associations were generally small in magnitude. With respect to the proposed hypotheses, H2a was not supported; H2b was partially supported; H3a was not supported; H3b was not supported; H3c was not supported; and H4 was partially supported.

**Figure 4:**
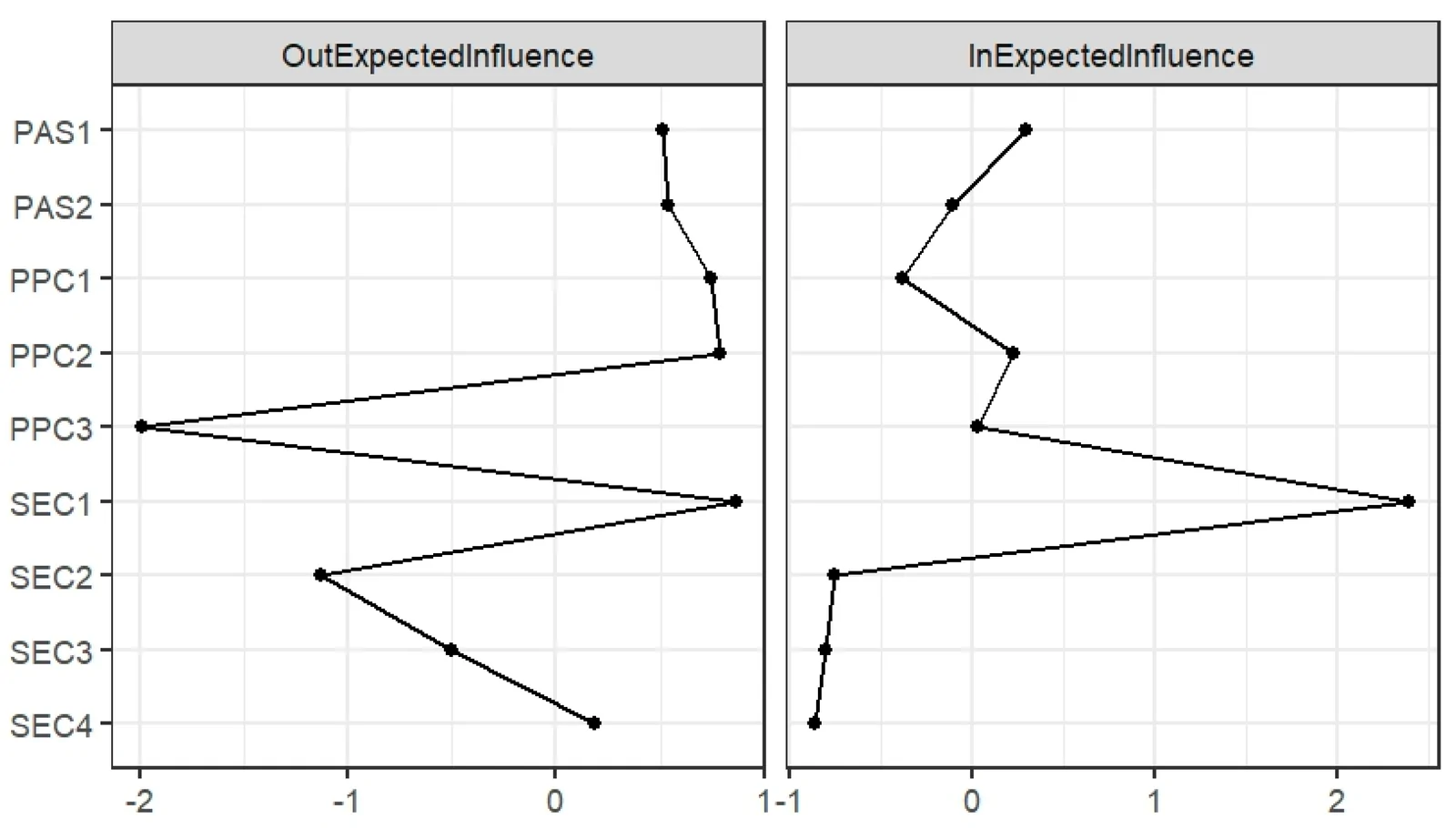
Node centrality within the W1 → W2 CLPN. SEC1: responsible decision-making; SEC2: relationship skills; SEC3: self-management; SEC4: social awareness; PAS1: choice making; PAS2: opinion exchange; PPC1: guilt induction; PPC2: love withdrawal; PPC3: authority assertion.

Figure 4 shows outEI and inEI. Within the CLPN, the component with the highest outEI is responsible decision-making, followed by love withdrawal. For the inEI, responsible decision-making ranks the highest, with choice making being the next. More details are shown in Supplementary Figure S2.

**Figure 5:**
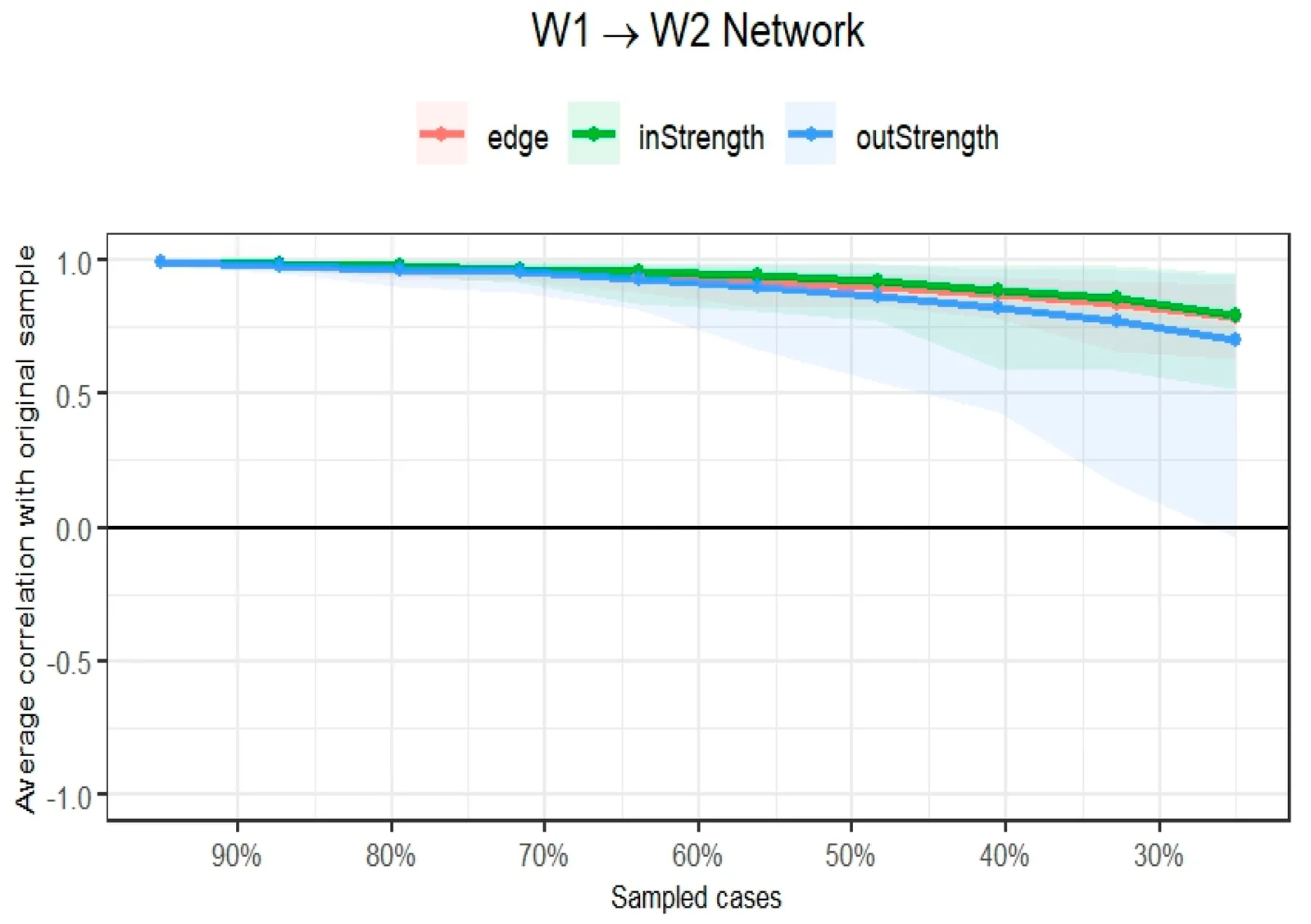
Case-dropping bootstrap stability of temporal edge strength and out-expected influence (outEI) and in-expected influence (inEI) centrality. Curves show correlations between estimates from subsamples and the original sample.

Moreover, temporal network metric coefficients showed a strong stability for edge strength (CS = 0.672), inEI (CS = 0.517), and outEI (CS = 0.439). The 95% confidence intervals (CIs) for edge, inEI, and outEI were relatively narrow, suggesting that the edges and centrality indicators were estimated with good precision (Figure 5).

### 3.4. Comparison Between Waves

At Wave 1, 25 of 36 possible edges were nonzero (density = 0.694), and the global strength was 3.657; at Wave 2, 26 edges were nonzero (density = 0.722), and the global strength was 3.802. A paired permutation-based Network Comparison Test (1000 permutations) indicated no significant difference in global strength between waves (S = 0.145, *p* = 0.129). The overall network structure differed modestly (M = 0.115, *p* = 0.027), although edge weights were highly correlated across waves (r = 0.985) and 23 nonzero edges were shared (Jaccard index = 0.821). After Holm correction, the PAS2–PPC2 edge differed significantly between waves (adjusted *p* = 0.036), indicating a weaker negative association at Wave 2 than at Wave 1. For the temporal model, repeated 10-fold cross-validation yielded a mean out-of-sample R^2^ of 0.287 across the nine Wave 2 outcomes (range = 0.089–0.410), indicating an acceptable-to-moderate predictive performance (see Supplementary Table S3 for full results).

## 4. Discussion

This two-wave study examined facet-specific prospective associations among parental autonomy support, psychological control, and adolescents’ SEC, further clarifying the differentiated prospective associations between parenting styles and adolescents’ SEC. The principal finding was that opinion exchange showed positive prospective associations with several SEC facets, whereas love withdrawal showed a negative prospective association with responsible decision-making. A smaller adolescent-to-parent path linked higher self-management with lower subsequent authority assertion. These findings extend the prior variable-level work by showing that associations were not uniform across parenting styles or SEC facets, but also underscore the necessity of attending to the associations among family and adolescents’ social adaptation skills.

The contemporaneous networks showed that different dimensions of parenting styles are associated with selected SEC facets after conditioning on the other included nodes. The negative contemporaneous association between love withdrawal and responsible decision-making is consistent with attachment-based accounts suggesting that conditional affection may be related to lower relational security and less autonomous decision-making (Bowlby, 1969; Freeman & Brown, 2001). There is a positive correlation between parents’ communication with their children and relationship skills, self-management ability, and social awareness, demonstrating that high levels of open communication are closely linked to strong self-regulation capabilities. These support SDT (Ryan & Deci, 2017; Bradshaw et al., 2025), which posits that the fulfillment of autonomy serves as the foundation for both intrinsic motivation and social integration. The cultivation of autonomy may provide opportunities related to the development of adolescents’ autonomous learning abilities and self-control skills.

Opinion exchange, love withdrawal, and social awareness showed relatively prominent cross-community connectivity in the contemporaneous networks. These three components influence the changes and connectivity relationships within the network, fundamentally highlighting that the core manifestation of interpersonal relationships lies in emotional security (Bowlby, 1969; Freeman & Brown, 2001). Opinion exchange reflects the quality and interaction patterns between adolescents and their parents, while love withdrawal indicates parental threats and control over adolescents’ values and behaviors. Closely linked to these are adolescents’ social awareness, which encompasses their understanding of others’ emotions. This provides micro-level evidence supporting the Family Systems Theory (Baptist & Hamon, 2022); only relational responsiveness can transcend educational boundaries to impact core pathways of adolescent development, particularly through adolescents’ comprehension of others’ behaviors and emotions (Y. Wang & Guo, 2024).

We further observed the prospective effects in CLPN, which are broadly consistent with SDT (Ryan & Deci, 2017). The clearest parent-to-adolescent prospective association linked a higher opinion exchange with higher self-management after three months. Within a parenting approach that respects children’s ideas and encourages mutual exchange of thoughts, adolescents need to consistently express their viewpoints (Cui et al., 2014; Y. Wang & Guo, 2024). Open parent–adolescent communication may provide adolescents with opportunities to practice negotiation, perspective-taking, and behavioral regulation, while also facilitating the internalization of parental expectations as personally endorsed standards. Such autonomous internalization is more likely than pressure-driven compliance to support sustained self-regulation (Ryan & Deci, 2017; Soenens & Vansteenkiste, 2010).

Notably, the pattern of contemporaneous regularized partial network relations did not necessarily map on to temporal network relations (Zainal & Newman, 2023/2024). A limited adolescent-to-parent prospective association was also observed. When adolescents demonstrate stronger self-management, parents may show lower authority assertion over the short term. This finding is consistent with previous work suggesting that adolescent characteristics may be prospectively related to subsequent parenting (van Meegen et al., 2024; J. Wang et al., 2023). For example, adolescent adjustment competences and parental psychological control may be prospectively associated with one another over time (Yu et al., 2021; Deng et al., 2024). Adolescents who regulate emotions and behavior effectively may be perceived as more predictable and capable of handling responsibility. Parents may consequently feel less need to enforce compliance through authority and may become more willing to negotiate or provide choices. This pattern challenges a strictly top-down model in which parents act and adolescents merely receive influence. Viewing parent and child characteristics as mutually shaping across repeated interactions is more consistent with transactional models of development (Sameroff, 1975). Nevertheless, most cross-lagged effects were small in magnitude and should therefore be interpreted cautiously.

Furthermore, responsible decision-making showed relatively prominent incoming and outgoing connectivity in the estimated temporal network. This capacity for responsible decision-making, a moral code for behavior, combines strong motivational power with high sensitivity. Therefore, this pattern suggests that responsible decision-making may warrant further investigation in confirmatory and intervention studies. Notably, love withdrawal, another pivotal parenting factor within outEI, underscores once again the critical role of parental emotional support in adolescent development.

The findings may be interpreted within the broader context of contemporary Chinese parenting, in which autonomy-supportive and controlling practices may coexist. The facet-level results suggest that these practices are not functionally uniform, indicating that broad parenting labels may conceal meaningful differences among specific practices (Xu et al., 2024). Parents may support adolescent participation and discussion in some domains while retaining stronger authority in others. In this context, the prominence of opinion exchange suggests that parent–adolescent communication may be particularly relevant.

## 5. Strengths, Limitations, and Future Research

This study has several strengths. First, the longitudinal design allowed prospective associations between parenting practices and adolescents’ SEC to be examined across time. Second, autonomy support and psychological control were considered simultaneously within a unified framework, allowing their differentiated associations with SEC to be compared. Third, the facet-level network approach moved beyond broad composite scores and enabled the identification of specific associations among parenting practices and individual SEC.

Several limitations should be acknowledged. First, the use of the Delaware Social-Emotional Competence Scale contrasts with the diverse scales available for measuring SEC in most of the existing literature. Future studies could employ more detailed dimensional classifications to explore internal correlations within this domain. Second, the reliance on self-reported data may introduce social desirability effects. The subsequent research should incorporate teacher-reported assessments of students’ SEC for enhanced representativeness. Third, the three-month interval between the two waves was designed to capture short-term prospective associations, but it limits the examination of longer-term temporal patterns. The two-wave design cannot separate stable between-person differences from within-person change or evaluate temporal stationarity across multiple intervals. Future studies should extend the follow-up period and include additional measurement waves to address these limitations. Finally, heterogeneity across regions, urbanicity, socioeconomic position, and family structures limits broad cultural generalization. Comparisons across regions, socioeconomic groups, rural and urban settings, and birth cohorts are needed to determine how the functions of autonomy support and psychological control vary within contemporary China.

## 6. Conclusions

This study used a two-wave cross-lagged panel network approach to examine facet-level associations between parenting styles and adolescents’ SEC. The findings primarily revealed facet-specific prospective associations from parenting practices to later SEC, together with limited evidence for an adolescent-to-parent association. Furthermore, responsible decision-making may warrant further investigation in intervention research. From a practical perspective, these findings may provide directions for future intervention research. Family-based programs may benefit from encouraging open parent–adolescent opinion exchange and reducing the reliance on the conditional withdrawal of affection, while school-based programs may provide more opportunities for adolescents to practice self-management and responsible decision-making. Overall, by moving beyond broad parenting and competence constructs, this study provides a more differentiated understanding of how specific family practices and adolescent SEC are prospectively intertwined during adolescence.

## Figures and Tables

**Table 1:** Descriptive statistics of networks components.

	Responsible Decision-Making	Relationship Skills	Self-Management	Social Awareness	Choice Making	Opinion Exchange	Guilt Induction	Love Withdrawal	Authority Assertion
Wave 1									
M	9.76	9.22	9.18	9.16	3.37	3.28	2.43	1.98	2.71
SD	1.43	1.63	1.59	1.50	0.77	0.94	0.92	0.99	1.19
Min	4	3	4	3	1	1	1	1	1
Max	12	12	12	12	5	5	5	5	5
Wave 2									
M	9.74	9.17	9.15	9.16	3.37	3.25	2.55	2.20	2.79
SD	1.52	1.68	1.67	1.52	0.82	0.97	0.97	1.07	1.20
Min	3	3	3	3	1	1	1	1	1
Max	12	12	12	12	5	5	5	5	5

Note: M, mean; SD, standard deviation; Min, minimum; Max, maximum. Responsible decision-making, Relationship skills, Self-management, and Social awareness were derived from the Delaware Social-Emotional Competency Scale; choice making, and opinion exchange were derived from the Psychological autonomy support Scale; guilt induction, love withdrawal, and authority assertion were derived from the Psychological control scale. All values represent raw scores.

**Table 2:** Strongest undirected edges of contemporaneous networks.

Measure	Undirected Edge Weight		
	W1	W2	Average
SEC1-SEC2	0.400	0.316	0.358
SEC1-SEC3	0.219	0.247	0.233
SEC2-SEC3	0.273	0.365	0.319
SEC1-SEC4	0.017	0.003	0.010
SEC2-SEC4	0.120	0.122	0.121
SEC3-SEC4	0.143	0.125	0.134
SEC2-PAS1	0.000	0.004	0.002
SEC4-PAS1	0.056	0.060	0.058
SEC1-PAS2	0.053	0.040	0.047
SEC2-PAS2	0.078	0.022	0.050
SEC3-PAS2	0.088	0.153	0.121
SEC4-PAS2	0.005	0.000	0.002
PAS1-PAS2	0.659	0.717	0.688
SEC2-PPC1	0.025	0.000	0.012
SEC3-PPC1	−0.020	0.000	−0.010
SEC4-PPC1	0.033	0.043	0.038
PAS1-PPC1	−0.029	−0.026	−0.028
PAS2-PPC1	−0.037	−0.071	−0.054
SEC1-PPC2	−0.049	−0.050	−0.050
SEC3-PPC2	−0.019	0.000	−0.010
SEC4-PPC2	0.000	0.009	0.004
PAS1-PPC2	−0.074	−0.076	−0.075
PAS2-PPC2	−0.124	0.000	−0.062
PPC1-PPC2	0.554	0.623	0.588
SEC2-PPC3	0.010	0.000	0.005
SEC4-PPC3	0.051	0.038	0.044
PAS1-PPC3	−0.009	−0.005	−0.007
PAS2-PPC3	−0.037	−0.068	−0.052
PPC1-PPC3	0.486	0.509	0.497
PPC2-PPC3	0.100	0.047	0.073

Note. SEC1: responsible decision-making; SEC2: relationship skills; SEC3: self-management; SEC4: social awareness; PAS1: choice making; PAS2: opinion exchange; PPC1: guilt induction; PPC2: love withdrawal; PPC3: authority assertion.

**Table 3:** Edge weight of CLPN.

Wave 1 to Wave 2 Measure	Directed Edge Weight
PPC1 → PPC3	0.295
PAS2 → SEC3	0.218
SEC1 → SEC2	0.207
PAS2 → PAS1	0.205
PAS1 → PAS2	0.178
SEC1 → SEC3	0.139
SEC3 → SEC1	0.106
PAS2 → SEC2	0.101
PPC1 → PPC2	0.100
PAS2 → SEC1	0.098
SEC2 → SEC3	0.093
PPC3 → PPC1	0.070
SEC2 → SEC1	0.067
PPC2 → PPC1	0.066
SEC2 → SEC4	0.060
PPC3 → SEC4	0.053
SEC4 → SEC2	0.043
PPC3 → PPC2	0.041
SEC3 → SEC2	0.033
SEC1 → PAS2	0.033
SEC3 → SEC4	0.029
PPC3 → SEC3	0.024
SEC1 → PAS1	0.016
SEC2 → PPC2	0.012
SEC2 → PAS2	0.009
SEC3 → PAS2	0.001
SEC1 → SEC4	0.001
PPC3 → PAS2	−0.002
SEC2 → PAS1	−0.007
SEC4 → PAS2	−0.007
SEC3 → PPC1	−0.010
SEC3 → PPC2	−0.026
PAS2 → PPC2	−0.031
PPC2 → PAS2	−0.034
PAS2 → PPC3	−0.035
PPC2 → SEC2	−0.047
SEC3 → PPC3	−0.050
PPC2 → PAS1	−0.059
PAS2 → PPC1	−0.060
PPC2 → SEC1	−0.071
PPC1 → PAS1	−0.075
PPC1 → PAS2	−0.091
PAS1 → PPC3	−0.129
PAS1 → PPC1	−0.144
PAS1 → PPC2	−0.171

Note. SEC1: responsible decision-making; SEC2: relationship skills; SEC3: self-management; SEC4: social awareness; PAS1: choice making; PAS2: opinion exchange; PPC1: guilt induction; PPC2: love withdrawal; PPC3: authority assertion.

## Data Availability

The data supporting the findings of this study are available from the corresponding author, Shuguang Zhang, upon reasonable request.

## Supplementary Materials

The following supporting information can be downloaded at: https://www.mdpi.com/article/10.3390/bs16101869/s1.
