# Supplementary material for: Prospective Associations Between Parenting Styles and Adolescents’ Social–Emotional Competence: A Cross-Lagged Network Analysis
Source: Behav Sci (Basel). 2026 Oct 10;16(10):1869. doi: 10.3390/bs16101869 (PMC13701355; doi:10.3390/bs16101869)
Supplement: Supplementary file 1 [file behavsci-16-01869-s001.zip › behavsci-4472064-supplementary.pdf]

Supplementary material for Behavioral Sciences

Table S1 Correlations among demographic variables and components.

	1	2	3	4	5	6	7	8	9	10	11	12	13	14	15	16	17	18	19	20	21	22
1.Gender	1																					
2.Age	-	1																				
3.Paternal level of education	-	-	1																			
4.Maternal level of education	-	-	-	1																		
5.Responsible decision making W1	0.01	-.111***	0.044	0.013	1																	
6.Relationship skills W1	0.016	-.110***	0.026	0.036	.556***	1																
7.Self-management W1	-.072*	-.089**	0.044	.061*	.454***	.505***	1															
8.Social awareness W1	0.04	-0.028	0.053	0.03	.196***	.252***	.282***	1														
9.Choice making W1	-0.019	-.071*	0.04	0.054	.206***	.190***	.227***	.143***	1													
10.Opinion exchange W1	-.073*	-.136***	.083**	.075*	.284***	.265***	.306***	.132***	.775***	1												
11.Guilt induction W1	-0.013	.110***	-0.007	-0.051	-.144***	-.087**	-.162***	0.031	-.434***	-.473***	1											
12.Love withdrawal W1	0	.135***	-0.001	-0.017	-.189***	-.112***	-.197***	-0.033	-.470***	-.513***	.805***	1										

13.Authority assertion W1	0.034	.076**	0.009	-0.008	-.117***	-0.053	-.125***	.059*	-.352***	-.381***	.725***	.608***	1									
14.Responsible decision making W2	0.016	-0.056	.059*	0.021	.421***	.329***	.326***	.102***	.178***	.242***	-.147***	-.184***	-.105***	1								
15.Relationship skills W2	-0.043	-0.012	.088**	0.036	.366***	.393***	.283***	.168***	.158***	.216***	-.084**	-.136***	-.066*	.551***	1							
16.Self-management W2	-.124***	-0.034	.077**	0.053	.345***	.344***	.434***	.155***	.203***	.284***	-.112***	-.155***	-.064*	.517***	.585***	1						
17.Social awareness W2	0.029	-.061*	.087**	.093**	.118***	.164***	.153***	.294***	0.026	0.047	0.036	0.014	.066*	.193***	.283***	.298***	1					
18.Choice making W2	-.079**	-0.031	0.025	0.03	.181***	.114***	.164***	.076**	.548***	.546***	-.388***	-.405***	-.308***	.228***	.251***	.324***	.138***	1				
19.Opinion exchange W2	-.093**	-.062*	0.039	.062*	.239***	.204***	.229***	.058*	.552***	.626***	-.402***	-.415***	-.322***	.282***	.283***	.383***	.094**	.822***	1			
20.Guilt induction W2	0.004	.089**	0.001	-0.028	-.132***	-.085**	-.169***	-0.018	-.418***	-.427***	.614***	.546***	.499***	-.172***	-.110***	-.150***	.082**	-.450***	-.484***	1		
21.Love withdrawal W2	-0.003	.084**	0.003	-0.014	-.145***	-.066*	-.179***	-0.032	-.400***	-.401***	.514***	.573***	.415***	-.172***	-.111***	-.147***	.080**	-.420***	-.438***	.833***	1	
22.Authority assertion W2	.061*	0.057	0.006	-0.015	-.131***	-.082**	-.190***	-0.01	-.348***	-.360***	.532***	.458***	.545***	-.161***	-.101**	-.148***	.072*	-.407***	-.441***	.775***	.653***	1

Note: W1, Wave1; W2, Wave2; Age, baseline age; Gender, Age, Paternal level of education and Maternal level of education were recoded as virtual variables. *: p<0.05; **: p<0.01; ***: p<0.001.

Note. Lower-triangular Pearson correlations are shown. W1 = Wave 1; W2 = Wave 2. * $p < 0.05$. ** $p < 0.01$. *** $p < 0.001$. Gender and parental education were coded numerically in the source analysis.

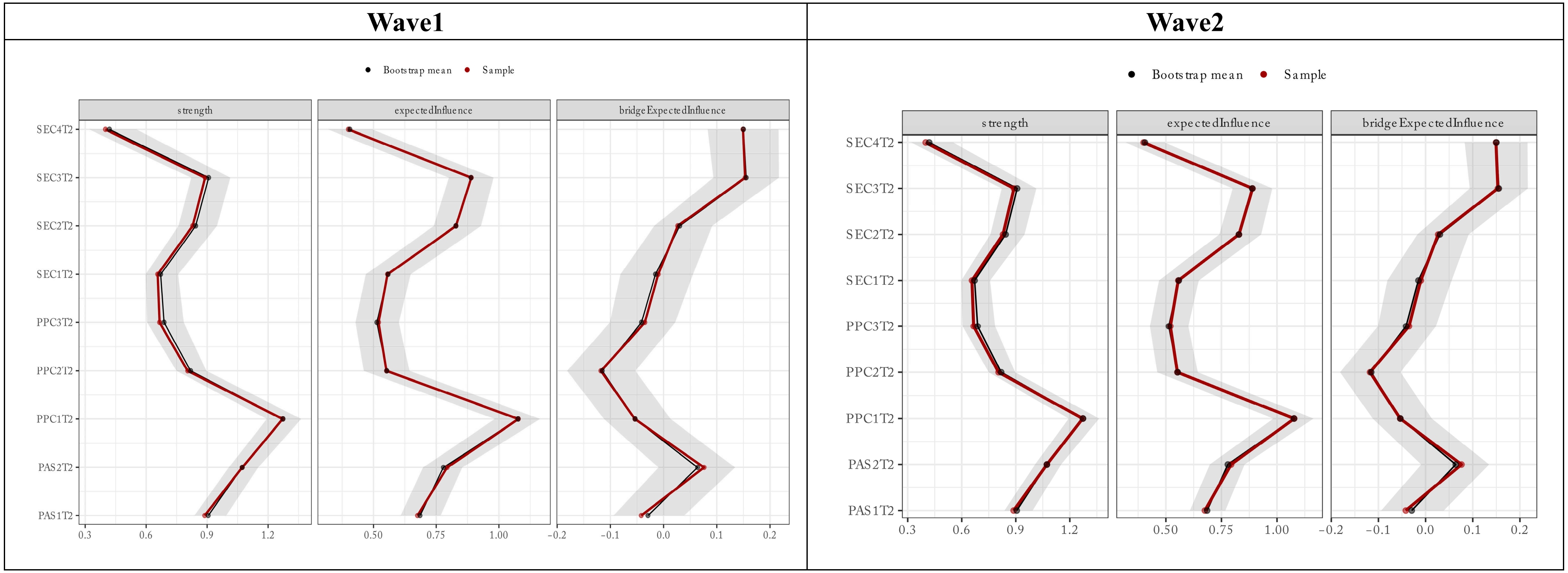

Figure S1

Case-dropping bootstrap stability of edge and bridge expected influence (bridge EI) at W1 and W2. Stability is evaluated by the correlations between bridge EI estimates from subsets of the data and estimates from the full sample.

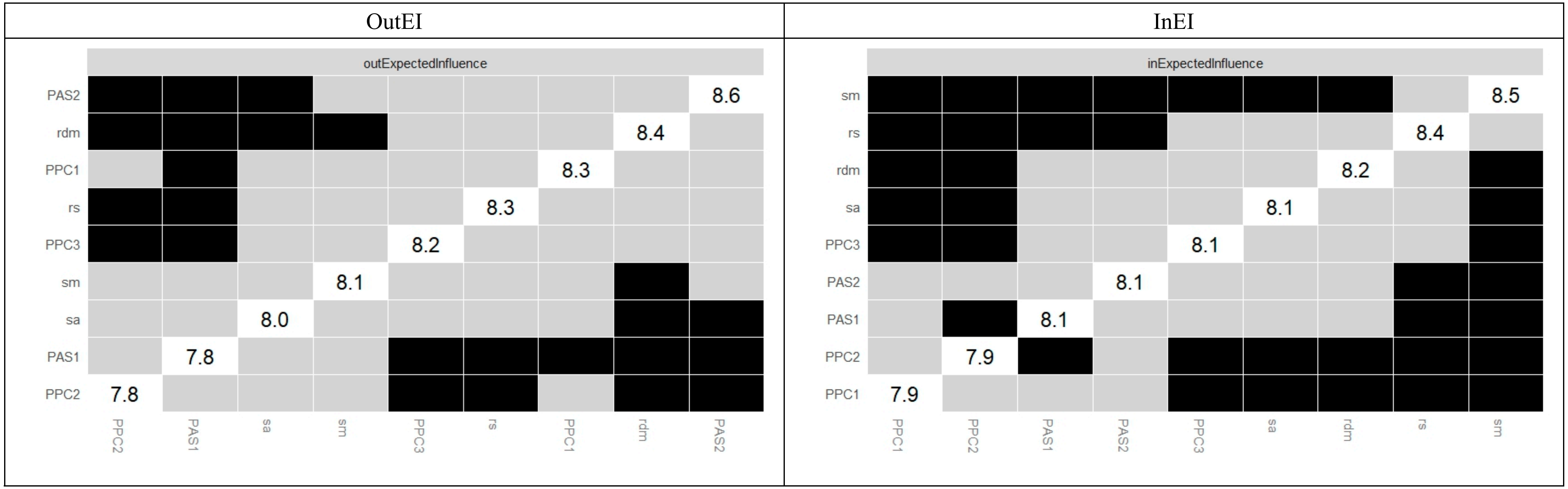

Figure S2. Out-expected influence (outEI) and in-expected influence (inEI) of CLPN.

Table S2. Longitudinal measurement invariance testing for the study constructs

Construct	Model	Robust χ^2	df	CFI	TLI	RMSEA	RMSEA 95% CI	SRMR	ΔCFI	ΔRMSEA	ΔSRMR	Supported
SEC	Configural	554.145	212	.935	.915	.040	.036–.044	.046	—	—	—	—
SEC	Metric	556.701	220	.936	.919	.039	.035–.043	.046	.001	-.001	.000	Yes
SEC	Scalar	575.080	228	.934	.920	.039	.035–.043	.046	-.002	-.000	.000	Yes

Construct	Model	Robust χ^2	df	CFI	TLI	RMSEA	RMSEA 95% CI	SRMR	ΔCFI	ΔRMSEA	ΔSRMR	Supported
SEC	Strict	596.481	240	.932	.922	.039	.035–.043	.047	-.002	-.000	.001	Yes
PAS	Configural	660.512	234	.963	.956	.044	.040–.048	.029	—	—	—	—
PAS	Metric	679.921	244	.963	.958	.043	.040–.047	.032	-.000	-.001	.003	Yes
PAS	Scalar	718.864	254	.960	.957	.044	.040–.048	.033	-.002	.000	.001	Yes
PAS	Strict	779.130	266	.956	.955	.045	.041–.049	.033	-.004	.001	.001	Yes
PPC	Configural	2605.748	561	.906	.895	.065	.063–.068	.045	—	—	—	—
PPC	Metric	2638.357	576	.906	.897	.064	.062–.067	.047	-.000	-.001	.003	Yes
PPC	Scalar	2689.638	591	.905	.899	.064	.061–.066	.047	-.001	-.000	.000	Yes
PPC	Strict	2776.024	609	.902	.898	.064	.062–.066	.048	-.003	.000	.000	Yes

Note. N = 1,167. Models were estimated using robust maximum likelihood (MLR), with residuals of corresponding items allowed to correlate across waves. Metric invariance constrained factor loadings; scalar invariance additionally constrained item intercepts; strict invariance additionally constrained residual variances. Invariance was judged supported when ΔCFI ≥ −.010 and ΔRMSEA ≤ .015; ΔSRMR ≤ .030 for metric invariance and ≤ .010 for scalar and strict invariance. All three constructs met the prespecified change-in-fit criteria through strict invariance. The SEC solution should nevertheless be interpreted cautiously because the Wave 1 latent correlation between responsible decision-making and self-management was 1.006, indicating a localized discriminant-validity concern.

Table S3. Paired Network Comparison Test across waves

Panel A. Global network comparison

Comparison	W1	W2	Test statistic	p	Interpretation
Nonzero edges, n	25	26	—	—	Descriptive
Network density	.694	.722	—	—	Descriptive

Comparison	W1	W2	Test statistic	p	Interpretation
Global strength	3.657	3.802	S = .145	.129	No significant difference
Network structure	—	—	M = .115	.027	Significant difference
Correlation of all 36 edge weights	.985	—	—	—	High cross-wave similarity
Jaccard overlap of nonzero edges	.821	—	—	—	23 shared edges

Note. The paired Network Comparison Test used 1,000 permutations, EBICglasso with $\gamma = .50$, weighted networks, and absolute edge weights for the global-strength test. S is the absolute difference in global strength; M is the maximum absolute difference between corresponding edge weights. The density and overlap statistics are descriptive and are not separate permutation tests.

Panel B. All edge-specific comparisons

Edge	W1 weight	W2 weight	W1 − W2	|Difference|	Raw p	Holm-adjusted p	Significant
PAS2–PPC2	-.121	-.006	-.115	.115	< .001	.036	Yes
SEC2–SEC3	.293	.360	-.068	.068	.081	1.000	No
PAS1–PAS2	.660	.726	-.067	.067	.005	.175	No
SEC3–PAS2	.092	.145	-.053	.053	.060	1.000	No
SEC1–SEC2	.393	.342	.051	.051	.201	1.000	No
SEC1–SEC3	.203	.252	-.049	.049	.230	1.000	No
PPC1–PPC2	.593	.638	-.045	.045	.106	1.000	No
PAS2–PPC3	-.026	-.065	.039	.039	.271	1.000	No
SEC1–PAS2	.068	.031	.038	.038	.191	1.000	No

Edge	W1 weight	W2 weight	W1 − W2	|Difference|	Raw p	Holm-adjusted p	Significant
SEC4–PPC2	.000	.036	-.036	.036	.015	.509	No
PPC1–PPC3	.473	.505	-.033	.033	.291	1.000	No
PAS2–PPC1	-.048	-.080	.032	.032	.358	1.000	No
PPC2–PPC3	.069	.038	.031	.031	.462	1.000	No
SEC2–PAS2	.043	.015	.028	.028	.338	1.000	No
SEC2–SEC4	.099	.126	-.026	.026	.567	1.000	No
SEC3–PPC2	-.025	.000	-.025	.025	.058	1.000	No
SEC4–PPC3	.049	.029	.021	.021	.609	1.000	No
SEC1–SEC4	.020	.002	.018	.018	.717	1.000	No
SEC4–PPC1	.022	.039	-.018	.018	.602	1.000	No
SEC2–PAS1	.000	.015	-.015	.015	.159	1.000	No
SEC1–PPC3	.000	-.014	.014	.014	.524	1.000	No
PAS1–PPC2	-.067	-.054	-.013	.013	.739	1.000	No
SEC1–PPC2	-.022	-.031	.009	.009	.718	1.000	No
SEC3–SEC4	.159	.152	.007	.007	.887	1.000	No
PAS1–PPC1	-.025	-.020	-.005	.005	.892	1.000	No
SEC2–PPC3	.005	.000	.005	.005	.665	1.000	No

Edge	W1 weight	W2 weight	W1 − W2	|Difference|	Raw p	Holm-adjusted p	Significant
PAS1–PPC3	-.021	-.018	-.004	.004	.901	1.000	No
SEC4–PAS1	.060	.061	-.002	.002	.970	1.000	No
SEC1–PAS1	.000	.000	.000	.000	1.000	1.000	No
SEC3–PAS1	.000	.000	.000	.000	1.000	1.000	No
SEC4–PAS2	.000	.000	.000	.000	1.000	1.000	No
SEC1–PPC1	.000	.000	.000	.000	1.000	1.000	No
SEC2–PPC1	.000	.000	.000	.000	1.000	1.000	No
SEC3–PPC1	.000	.000	.000	.000	1.000	1.000	No
SEC2–PPC2	.000	.000	.000	.000	1.000	1.000	No
SEC3–PPC3	.000	.000	.000	.000	1.000	1.000	No

Note. Edges are undirected regularized partial correlations and are ordered by decreasing absolute cross-wave difference. Raw p values were calculated from the saved 1,000-permutation edge-difference distributions using the plus-one correction; Holm-adjusted p values are those returned by the NCT object for all 36 edge tests. Only PAS2–PPC2 remained significant after Holm correction. Node abbreviations: SEC1 = responsible decision-making; SEC2 = relationship skills; SEC3 = self-management; SEC4 = social awareness; PAS1 = choice making; PAS2 = opinion exchange; PPC1 = guilt induction; PPC2 = love withdrawal; PPC3 = authority assertion.
